# A Computational Study of the Electrophysiological Substrate in Patients Suffering From Atrial Fibrillation

**DOI:** 10.3389/fphys.2021.673612

**Published:** 2021-07-08

**Authors:** S. Pagani, L. Dede', A. Frontera, M. Salvador, L. R. Limite, A. Manzoni, F. Lipartiti, G. Tsitsinakis, A. Hadjis, P. Della Bella, A. Quarteroni

**Affiliations:** ^1^MOX-Department of Mathematics, Politecnico di Milano, Milan, Italy; ^2^Department of Arrhythmology, San Raffaele Hospital, Milan, Italy; ^3^Institute of Mathematics, EPFL, Lausanne, Switzerland

**Keywords:** atrial fibrillation, numerical simulation, cardiac electrophysiology, mathematical models, arrhythmia

## Abstract

In the context of cardiac electrophysiology, we propose a novel computational approach to highlight and explain the long-debated mechanisms behind atrial fibrillation (AF) and to reliably numerically predict its induction and sustainment. A key role is played, in this respect, by a new way of setting a parametrization of electrophysiological mathematical models based on conduction velocities; these latter are estimated from high-density mapping data, which provide a detailed characterization of patients' electrophysiological substrate during sinus rhythm. We integrate numerically approximated conduction velocities into a mathematical model consisting of a coupled system of partial and ordinary differential equations, formed by the monodomain equation and the Courtemanche-Ramirez-Nattel model. Our new model parametrization is then adopted to predict the formation and self-sustainment of localized reentries characterizing atrial fibrillation, by numerically simulating the onset of ectopic beats from the pulmonary veins. We investigate the paroxysmal and the persistent form of AF starting from electro-anatomical maps of two patients. The model's response to stimulation shows how substrate characteristics play a key role in inducing and sustaining these arrhythmias. Localized reentries are less frequent and less stable in case of paroxysmal AF, while they tend to anchor themselves in areas affected by severe slow conduction in case of persistent AF.

## 1. Introduction

Atrial fibrillation (AF) is the most prevalent cardiac arrhythmia worldwide, with elevated morbidity and mortality risks associated (see e.g., Kannel et al., 1998; Chugh et al., 2014). It is characterized by sequential irregular electrical activations, leading to ineffective atrial contraction. Despite substantial research efforts, the mechanisms underlying AF are not yet completely understood. Different theories have been proposed along the last decades to explain initiation, maintenance and progression of AF over time, such as the wavelet theory (Moe, 1962), the focal atrial activities (Jaïs et al., 1997), the driver domains (Haissaguerre et al., 2014) or the ganglia and autonomic system (Chen and Tan, 2007), just to mention a few.

AF classification is based on event duration and spontaneous termination, as reported in the 2020 ESC Guidelines in Hindricks et al. (2020). In particular, paroxysmal AF (PAF) is defined as an episode that terminates either spontaneously or with cardioversion within 7 days of onset, while persistent AF (PsAF) is referred to an episode that is continuously sustained beyond 7 days. The clinical factors behind the distinction between these two AF forms are not fully understood yet. In paroxysmal AF, the role of specific triggers localized in the pulmonary veins has been universally recognized starting from the work of Haissaguerre et al. (1998). However, a debate is ongoing about the characterization of the electrical and structural substrate responsible for the different duration and spontaneous termination of AF forms, as well as on the key factors behind the transition from PAF to PsAF. The transition has been often associated with the progression of left atrium (LA) remodeling, either anatomical or electrical, leading to a greater probability of AF recurrence; evidences in this direction have been shown, e.g., in Tieleman et al. (1998); Lu et al. (2008); Nattel et al. (2014).

Electrophysiological studies (EPSs), combined with radiofrequency catheter ablation, nowadays provide a well-established procedure to treat AF patients. In this respect, an EPS provides a detailed characterization of the electrophysiological properties of the LA, unvealing possible targets of ablation, such as complex fractionated atrial electrograms, low-voltage and slow conducting areas, and pivot points (see e.g. Cheniti et al., 2018). Structural defects of the atria, in the form of fibrosis, can be also identified using medical imaging (see Marrouche et al., 2014). Areas of patchy fibrosis retrieved from late-gadolinium enhanced magnetic resonance imaging (LGE MRI) have been associated with arrhythmic activity. However, functional properties, which are strictly related to the electrical remodeling, cannot be investigated by means of LGE MRI, thus removing a fundamental element to understand the mechanisms of AF.

In recent years, the use of computational models, parameterized with data extracted from medical imaging, has been proposed to balance the aforementioned lack of information. These computational studies exploit the numerical simulation of electrophysiology mathematical models, which consist of a coupled system of partial and ordinary differential equations, such as the modomain equation to describe the transmembrane potential and the so-called ionic models for the description of ionic species dynamics (see e.g., Franzone et al., 2014; Quarteroni et al., 2019). In these computational studies, patient-specific MRI-based atrial models were employed to analyze the stabilization of localized reentries in fibrotic areas (see e.g., McDowell et al., 2015; Zahid et al., 2016; Boyle et al., 2018; Cochet et al., 2018; Roy et al., 2020), which are then marked as possible patient-specific ablation targets (Boyle et al., 2019). However, mathematical models of LA electrophysiology need to be complemented by several additional patient-specific and spatially heterogeneous data, namely parameters and functional data, such as the conductivity tensor, the fiber orientation, and the ionic channel coefficients.

As from imaging data it is only possible to calibrate few parameters or infer a limited amount of patient-specific information, this leaves several other crucial ones as incomplete or totally undetermined. In these cases, as the calibration process would reveal to be ineffective, it is customary to select parameters from the literature or to make simplifying assumptions; however, the overall computational pipeline might yield numerical results that prove to be ineffective in order to shed light on the real patient-specific AF mechanisms, despite the reliability of the aforementioned physics-based models.

In this study, we propose a novel model parametrization based on conduction velocities, estimated from invasive high-density catheter mapping data, to numerically simulate both paroxysmal and persistent AF induction and sustainment in the LA. Specifically, our LA electrophysiology mathematical model couples the monodomain equation with the Courtemanche-Ramirez-Nattel (CRN) ionic model, which is particularly suitable for the human LA (see Courtemanche et al., 1998). A pipeline for the integration of invasive mapping data was firstly presented in Corrado et al. (2018) and validated against controlled-paced rhythms. Recently, in Lim et al. (2020) a new parametrization based on bipolar voltage maps was proposed and patient-specific AF simulations were used to identify targets of ablation. However, bipolar voltage measurements are extremely sensitive to the position of the two electrodes, and the definition of the optimal cutoff values among scarring tissue, border zone, and healthy tissue (see e.g., Kapa et al., 2014) is still subject to discussion. The development of a model parametrization driven by conduction velocities might provide a more detailed description of both structural and electrical remodeling, which might ultimately help to identify localized reentries' mechanisms. In this work, we consider patient-specificic mapping data coming from one paroxysmal and one persistent case, respectively, to compare their localized reentries' pattern. Furthermore, the model encodes the effects of electrical remodeling in fibrotic regions. This is achieved by modifying the ionic coefficients associated to the transient outward current, the ultrarapid delayed rectifier current and the L-type calcium current in areas of slow-conduction. Finally, to include all the basic factors promoting AF, we consider the presence of a high-frequency trigger from one of the pulmonary veins. The numerical simulations, performed once the mathematical model has been discretized with respect to both space and time, allow to study localized reentries' formation, their sustainment and their relationship with the substrate characteristics. Compared to the previously mentioned MRI-based atrial models, the proposed new model parametrization relying on conduction velocity data is able to capture the dynamics of a wandering rotor along mild slow conduction areas, which are usually not captured when using models based on macro-regions with homogeneous electrical properties. Consistently, the model parametrization manages to simulate the formation of anchor points in areas affected by severe fibrosis, but also to distinguish between functional lines of block in areas with high heterogeneity and stable anchor points. These findings might further improve the identification of possible patient-specific ablation targets from numerical simulations, as currently done by Boyle et al. (2019) and Lim et al. (2020).

The paper is organized as follows. In section 2, we summarize the basic factors promoting atrial fibrillation. In section 3, we present the mathematical model we use to simulate cardiac electrophysiology. In section 4, we discuss the pipeline for the approximation of conduction velocities starting from invasive mapping data. Finally, in section 5 we integrate the conduction velocity data into the mathematical model, by designing a new model parametrization that takes into account all the basic factors promoting AF. A presentation of the obtained numerical results, together with, a brief description of the computational setting, and a numerical comparison between a paroxysmal and a persistent case, are reported in section 6, followed by the Limitations of the study and our Conclusions.

## 2. Factors promoting AF

AF results from a series of functional and structural factors, generating the substrate for localized reentrant circuits induction and sustainment. In these circuits, the electrical activation is characterized by: (i) a rotation of the wavefront around a structural obstacle, under the form of either a fixed anchoring point or a line of block, arising in patchy fibrotic tissue; (ii) a center of rotation (rotor) that travels along functional lines of block.

**Figure 1:**
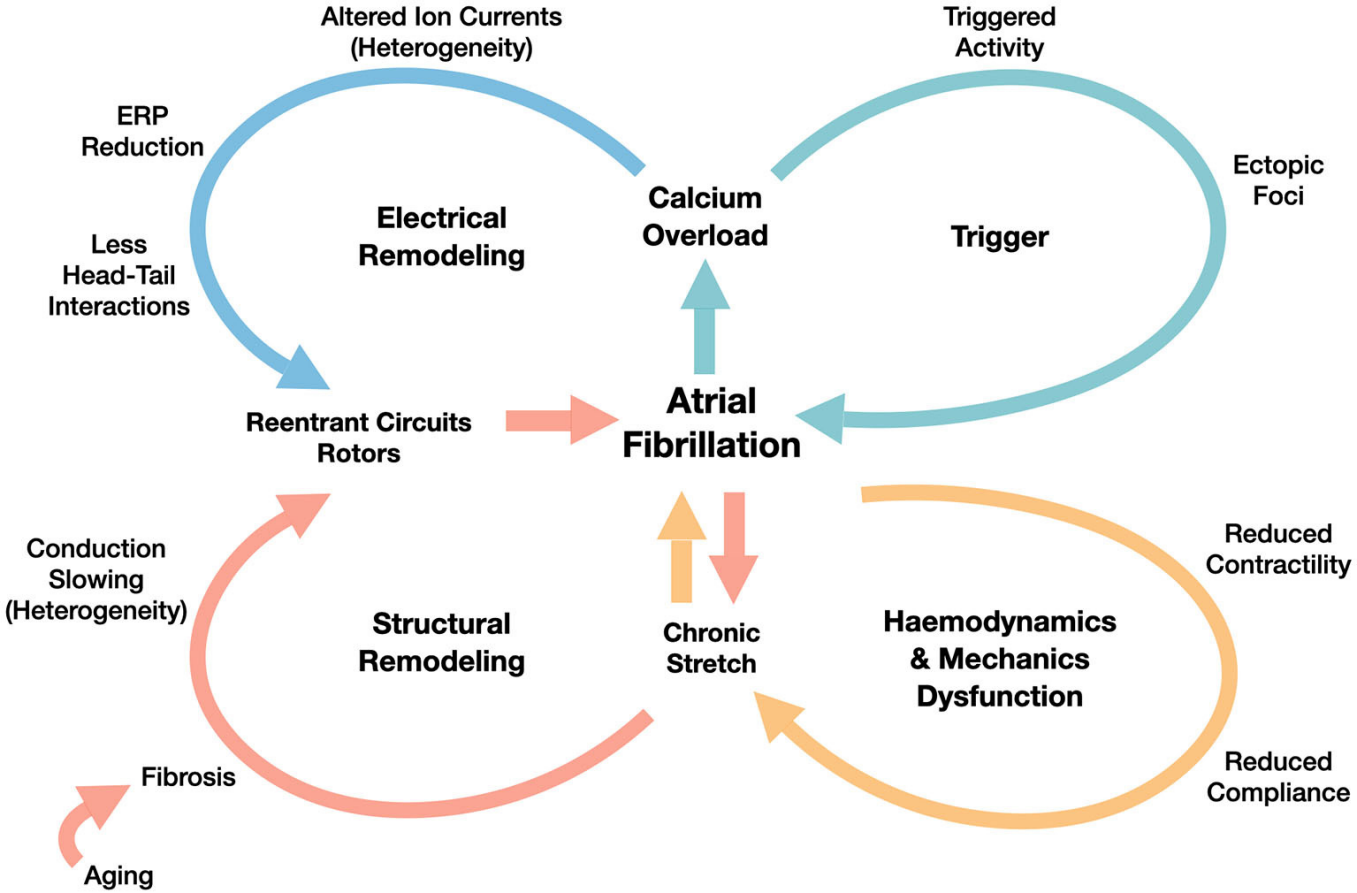
Overview of AF mechanisms generated by the interplay between functional (electrical remodeling and triggering) and structural factors (structural remodeling generated by the hemodynamics and mechanics dysfunction). We refer to Schotten et al. (2011) for the details of those mechanisms.

Following the scheme proposed in Schotten et al. (2011) (revised in Figure 1) and in Nattel et al. (2008), the basic factors promoting AF are the following ones:

1. *trigger activity*, which leads to the AF initiation through ectopic beats, that are mainly originated in pulmonary veins, as shown in Haissaguerre et al. (1998);
2. *electrical remodeling*, that includes shortening of the effective refractory period (ERP) and of the action potential duration (APD), which is also associated with the increase of the stimulation rate (rate adaptation). Electrical remodeling has been directly associated in Courtemanche et al. (1999) to changes in specific ionic currents, such as transient outward potassium current, L-type Calcium current and ultrarapid delayed rectifier current;
3. *structural remodeling*, characterized by defects induced by the formation of fibrosis and resulting in enhanced conduction heterogeneity in the tissue (conduction velocities are in the range [0−200] cm/s);
4. *hemodynamics and mechanics dysfunction*, firstly driven by the decrease of the atria contractility properties, yielding a decrease of the atria compliance, too.

The continuous interactions among the former four factors might explain AF progression from paroxysmal, to persistent, and finally to permanent. The initial, but unsustained episodes might accelerate the electrical remodeling and the worsening of the structural remodeling, generated by altered strains in the myocardium.

## 3. Mathematical model

The propagation of the electrical signal in the cardiac tissue is a multiscale process linking the microscale (ion channels dynamics) to the macroscale (atria tissue conductivity). In pathological conditions, this process across the scales is made even more complex by the strong heterogeneity of the tissue, due to the presence of fibrotic regions and electrical remodeling. A mathematical model that is able to describe the AF mechanisms should encode all this information.

With this in mind, we adopt the monodomain equation (Potse et al., 2006; Franzone et al., 2014), a time-dependent nonlinear diffusion-reaction partial differential equation (PDE) that describes the transmembrane potential dynamics at the tissue level, coupled with the CRN ionic model introduced in Courtemanche et al. (1998), which characterizes the dynamics of the ionic species concentrations and the ionic channels at the cellular level, modeling the behavior of the single cardiomyocyte of the atria.

The coupled electrophysiological model reads as follows:

(1) $$\begin{array}{l} \{\begin{array}{ll} \chi \left[C_{m}\frac{\partial u}{\partial t}+I_{\text{ion}}\left(u,w,c\right)\right]=\nabla \cdot \left(D\nabla u\right)+I_{\text{app}}\left(t\right) & \text{in }\Omega_{0}\times \left(0,T\right),\\ \frac{\partial w}{\partial t}-H\left(u,w\right)=0 & \text{in }\Omega_{0}\times \left(0,T\right),\\ \frac{\partial c}{\partial t}-G\left(u,w,c\right)=0 & \text{in }\Omega_{0}\times \left(0,T\right),\\ D\nabla u\cdot n=0 & \text{on }\partial \Omega_{0}\times \left(0,T\right),\\ u=u_{0} & \text{in }\Omega_{0}\times \left\{0\right\}, \end{array} \end{array}$$

where *u* represents the normalized transmembrane potential, *t* is the time variable, vectors ***w*** = {*w*_1_, *w*_2_, ..., *w*_*k*_} and ***c*** = {*c*_1_, *c*_2_, ..., *c*_*m*_} define *k* = 15 gating variables and *m* = 5 concentrations of specific ionic species (such as Ca^2+^, Na^+^ and K^+^), respectively. Here, $\Omega_{0}\subset {\mathbb{R}}^{3}$ is the fixed computational domain (left atria, simplified to a thin layer of tissue), with outward unit normal ***n*** to the boundary ∂Ω. Physical coefficients, such as the total membrane capacitance *C*_*m*_ and the area of cell membrane per tissue volume χ, complete the monodomain model, together with the diffusivity tensor

(2) $$\begin{array}{l} D=\sigma_{l}{\text{f}}_{0}⊗{\text{f}}_{0}+\sigma_{t}{\text{s}}_{0}⊗{\text{s}}_{0}+\sigma_{n}{\text{n}}_{0}⊗{\text{n}}_{0}. \end{array}$$

The tensor ***D*** = ***D***(**x**) encapsulates the fibers-sheets-crossfibers structure expressed by the vector fields **f**_0_, **s**_0_ and **n**_0_, respectively. This architecture, revealed by using submillimeter diffusion tensor magnetic resonance imaging in Pashakhanloo et al. (2016), encodes the structural contributions to atrial activation pattern: the conductivities σ_*l*_, σ_*t*_ and σ_*n*_ regulate the anisotropy in the signal propagation along the directions **f**_0_, **s**_0_ and **n**_0_, as shown in Roberts et al. (1979) and Zhao et al. (2012). $I_{\text{app}}\left(t\right)$ is an external applied current, which can model the complex physiological activation (from the sinoatrial node to the fast conduction system of the Banchmann's bundle) or any arbitrary pacing sequence. Finally, the ionic current $I_{\text{ion}}\left(u,w,c\right)$ is the nonlinear reaction terms coupling the cellular scale (micro) to the tissue one (macro). A Neumann boundary condition is applied all over the boundary, under the simplifying assumption of electrically isolated domain, as done, e.g., in Potse et al. (2006).

To solve system (1) we need to discretize it both in space and in time. We consider a fine hexaedral partition $T_{h}$ of the LA volume Ω_0_ in hexahedra. Here, the subscript *h* refers to the average size of the hexahedra in the computational mesh. We apply the Galerkin-Finite Element (FE) method, over the finite dimensional space *X*_*h*_ ⊂ *X*(Ω_0_), to numerically discretize the monodomain problem (1) in space. For the time discretization, we introduce the discrete times *t*^*n*^ = *nΔt*, *n* = 0, …, *N*_*t*_−1, which partition the time interval (0, *T*) in *N*_*t*_ evenly spaced subintervals of length Δ*t* and we adopt Backward Difference Formulae (BDF) scheme, introduced in Curtiss and Hirschfelder (1952). Finally, for the treatment of the nonlinear term and the ionic model, we adopt a segregated approach in which the ionic model advances in time first, in each node of the mesh, and then the updated values of both gating and concentration variables are used for the time advancement of the transmembrane potential in the monodomain model, as shown in Pagani et al. (2018). We refer the interested reader to Colli Franzone and Pavarino (2004), Colli Franzone et al. (2005), Potse et al. (2006), and Quarteroni et al. (2019) for the details of the numerical approximation.

In this setting, the discretized ionic model yields a system of ODEs, which indirectly depends on the space variable through the transmembrane potential at each time step. By denoting with ${\text{u}}_{h}^{n}$, ${\text{w}}_{h}^{n}$, and ${\text{c}}_{h}^{n}$ the transmembrane potential, the gating variables and the ionic concentrations approximated by the FE method at time *t*^*n*^, respectively, the fully-discrete formulation of the ionic model can be written as follows:

(3) $$\begin{array}{l} \{\begin{array}{ll} \frac{\alpha_{\text{BDF}}{\text{w}}_{h}^{n+1}-{\text{w}}_{h,\text{BDF}}^{n}}{\Delta t}=\text{H}\left({\text{u}}_{h,\text{BDF}}^{n},{\text{w}}_{h,\text{EXT}}^{n+1}\right) & n=0,...,N_{t}-1,\\ \frac{\alpha_{\text{BDF}}{\text{c}}_{h}^{n+1}-{\text{c}}_{h,\text{BDF}}^{n}}{\Delta t}=\text{G}\left({\text{u}}_{h,\text{BDF}}^{n},{\text{w}}_{h,\text{EXT}}^{n+1},{\text{c}}_{h,\text{EXT}}^{n+1}\right) & n=0,...,N_{t}-1,\\ {\text{w}}_{h}^{0}={\text{w}}_{0,h},\\ {\text{c}}_{h}^{0}={\text{c}}_{0,h}. \end{array} \end{array}$$

The fully-discretized formulation of the Monodomain equation reads as: for *n* = 0, ..., *N*_*t*_−1, find ${\text{u}}_{h}^{n+1}$ such that:

(4) $$\begin{array}{l} \text{M}\frac{\alpha_{\text{BDF}}{\text{u}}_{h}^{n+1}-{\text{u}}_{h,\text{BDF}}^{n}}{\Delta t}+\text{A}\left(D\right){\text{u}}_{h}^{n+1}+{\text{I}}_{\text{ion}}\left({\text{u}}_{h,\text{EXT}}^{n+1},{\text{w}}_{h}^{n+1},{\text{c}}_{h}^{n+1}\right)\\ \;={\text{I}}_{\text{app}}\left(t^{n+1}\right). \end{array}$$

Here, the matrices and vectors arising from the FE discretization are denoted by **M** (mass matrix), **A** (stiffness matrix), **I**_ion_ (discretized ionic current term) and **I**_app_ (discretized applied current term). The vectors ${\text{u}}_{h,\text{BDF}}^{n}$, ${\text{u}}_{h,\text{EXT}}^{n+1}$, ${\text{w}}_{h,\text{BDF}}^{n}$, ${\text{w}}_{h,\text{EXT}}^{n+1}$, ${\text{c}}_{h,\text{BDF}}^{n}$, ${\text{c}}_{h,\text{EXT}}^{n+1}$ are extrapolations of the same order of the selected BDF scheme. There are several strategies in literature for the treatment of the nonlinear term $I_{\text{ion}}\left(\cdot ,\cdot ,\cdot \right)$. Here, we adopt the so-called single variable interpolation (SVI) approach (see e.g., Pathmanathan et al., 2011, 2012; Krishnamoorthi et al., 2013).

For all the numerical simulations we consider a BDF approximation of order 3 as time advancing scheme, and linear Finite Element for space discretization. We refer the interested reader to Quarteroni et al. (2010).

## 4. Estimation of the conduction velocity

Electroanatomical maps acquired during an EPS reveal the presence of areas of low voltage and slow conduction, which can be directly associated with fibrotic tissue. An automatic and detailed characterization of these areas can be achieved by a numerical approximation of the conduction velocity (CV) vector field from activation maps acquired during sinus rhythm (SR). The CV vector field depicts the magnitude and the direction of wavefront propagation, quantifying the heterogeneity in the cardiac tissue conduction properties. In this work, we aim at integrating this source of information into model (1) to study potential mechanisms for localized reentries' induction and sustainment through numerical simulations. Several methods have been proposed for CV estimation, such as triangulation techniques, finite difference techniques or polynomial surface fitting (for a complete review see Cantwell et al., 2015). In this work, we follow the last approach by adapting the pipeline for the numerical approximation of CV, which was developed in Frontera et al. (2020) for the left ventricle and designed starting from the work of Dallet et al. (2018) and Roney et al. (2019b).

**Figure 2:**
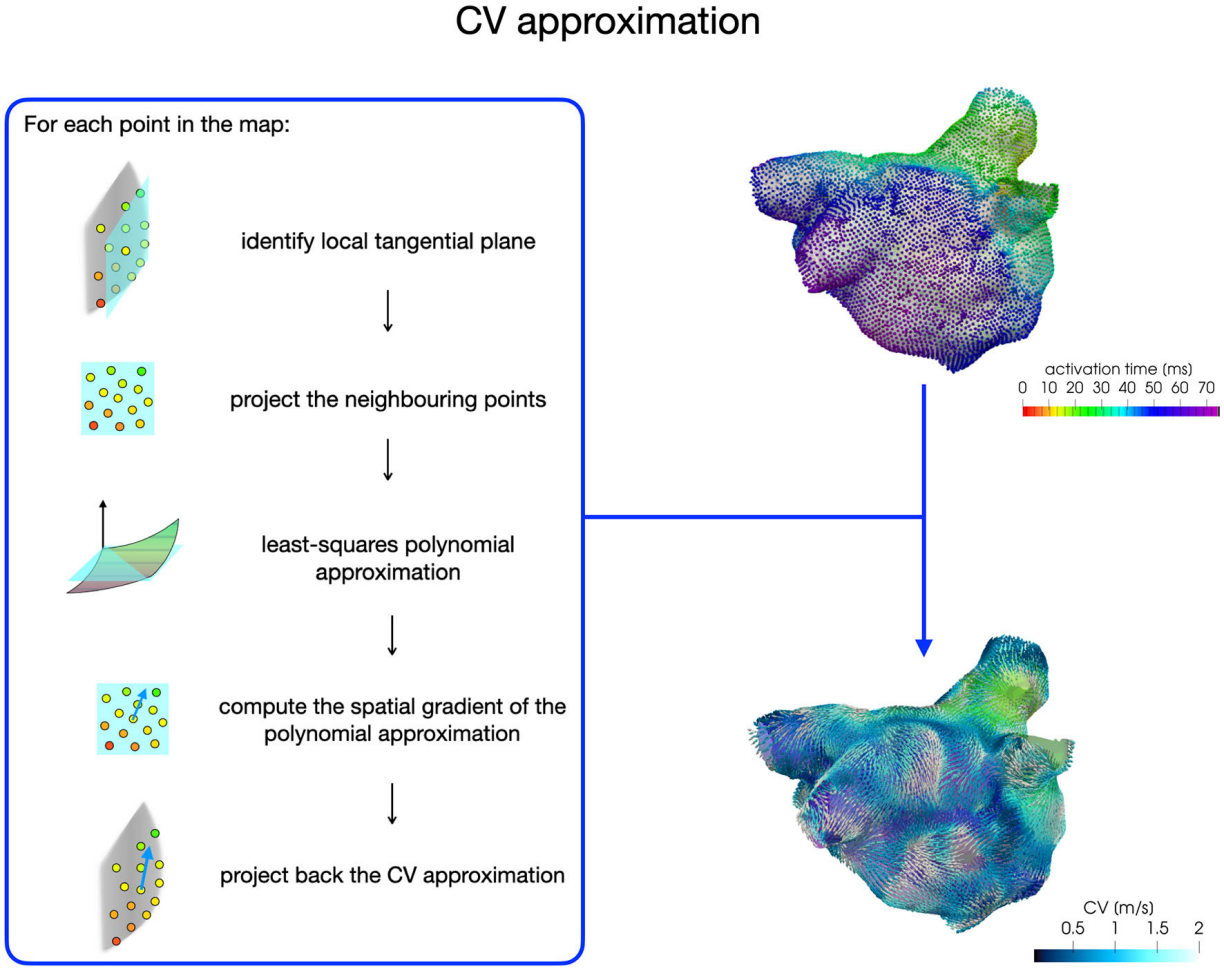
CV vector approximation procedure based on a least-squares approach.

For each patient, the CV vector approximation at each point of the map is obtained with a least-squares approach based on activation times acquired on a local patch (1 cm x 1 cm) of the map (see Figure 2). First, we build a local tangential plane, onto which we project the neighboring points belonging to the patch. Then, we compute the coefficients of a polynomial of degree two with a least-square approximation based on local activations and projected anatomical data. Finally, we compute the spatial gradient of the polynomial approximation, which is then projected back onto the LA map to reconstruct the three-dimensional CV vector. We consider CVs in the range [0,200] cm/s for the construction of the model parametrization.

In this work we start from two SR activation maps of the LA, acquired by the Arrhythmia Unit at San Raffaele Hospital, of a paroxysmal and a persistent patient, respectively. High-density electroanatomical maps were performed using Rhythmia (Boston Scientific) 3D mapping system and the Orion™ mapping catheter, which have an interelectrode spacing of 2.5 mm, acquiring more than 5, 000 signals on the atrial surface.

Should detailed LGE images be available, an approximation of conduction velocities could be also derived from pixel intensity, as recently proposed in Jang et al. (2019) and Aronis et al. (2020) for the ventricles. This procedure, however, aims at capturing differences in tissue conduction properties, rather than recovering the patient's actual conduction velocity. The possibility of integrating this source of information with activation time data would improve the accuracy of conduction velocity estimates, as well as the integration of additional data on the position and the shape of the mapping catheter (see e.g., Verma et al., 2018). We remark that our model parameterization, presented in the next section, can take advantage of any conduction velocity estimation technique, starting from the most adopted ones reviewed in Cantwell et al. (2015), up to new techniques, such as the streamline-based method of Good et al. (2020), the two-stage technique based on the depolarization pattern reconstruction shown in Nagel et al. (2019), the back-propagation parameter estimation procedure proposed in Pheiffer et al. (2017), or physics-informed neural networks applied to cardiac activation mapping in Sahli Costabal et al. (2020).

## 5. Integrating data into the model

To numerically simulate AF, we define a new model parametrization that includes the basic factors reviewed in section 2: trigger activity, electrical remodeling and structural substrate characterization. In this section we show how we, respectively parametrize the applied current, some coefficients of the ionic model, governing the conduction and refractory properties, and the heterogeneous diffusivity tensor. This new model parametrization is based on conduction velocity data, whose approximation from patient's activation maps is discussed in section 4.

The applied current $I_{\text{app}}\left(t\right)$ is parametrized to describe both the physiological activation sequence of the LA and the trigger activity from the pulmonary veins. We directly encode the complex and yet not well understood mechanism behind trigger activity in this term, which enables to artificially mimic afterdepolarizations that follow the upstroke of an action potential, whether these are early afterdepolarizations (EADs) or delayed afterdepolarizations (DADs). We also do not model the autonomic nerve activity, which can be one of the mechanisms behind the trigger activity, as shown in Chen and Tan (2007).

For the physiological baseline activation, we employ three impulses placed at spheres $S\left({\text{x}}_{p},r_{p}\right)$ of radius *r*_*p*_ and centered at points **x**_*p*_, *p* = 1, 2, 3, with a prescribed frequency *f*_*app*_ and duration *t*_*app*_

$$I_{\text{app}}\left(t\right)=\overset{3}{\underset{p=1}{\sum }}I_{\text{app}}\left(t;\text{x}\in S\left({\text{x}}_{p},r_{p}\right),f_{app},t_{app}\right).$$

Here, **x**_*p*_ are approximatively located in correspondence of the main inter-atrial connections: the Bachmann's Bundle (BB), the upper part of the Fossa Ovalis (FO) and the Coronary Sinus Musculature limbs (CSM) (for a detailed characterization, see Sakamoto et al., 2005). The impulses located in the FO and the CMS are delayed, with respect to the BB impulse, by 10 and 20 ms, respectively (see e.g., Piersanti et al., 2020).

For the trigger activity, we superimpose to the baseline activation a cubic-shaped impulse of length *l*_*p*_, centered at a point **x**_*t*_, with a given high frequency $f_{app}^{trig}$ and duration $t_{app}^{trig}$, under the form

(5) $$\begin{array}{l} I_{\text{app}}\left(t\right)=\overset{3}{\underset{p=1}{\sum }}I_{app}\left(t;\text{x}\in S\left({\text{x}}_{p},r_{p}\right),f_{app},t_{app}\right)\\ \;+I_{\text{app}}\left(t;\text{x}\in C\left({\text{x}}_{t},l_{p}\right),f_{app}^{trig},t_{app}^{trig}\right). \end{array}$$

Here, **x**_*t*_ is located in the lumen of one of the pulmonary veins, as measured in Haissaguerre et al. (1998). This modeling choice aims at reproducing a spontaneous induction phenomenon and neither the clinical one obtained through a stimulation protocol nor the artificial numerical one obtained by the superimposition of a planar wave. To encode in the model the local CV changes characterizing the diseased electrical substrate, we consider a diffusivity tensor under the form (2), where σ_*l*_(**x**), σ_*t*_(**x**) and σ_*n*_(**x**) are heterogeneous over Ω_0_ and parametrized following the approach presented in Costa et al. (2013). In particular, we model the electric conductivity coefficient along fibers using the following law:

$$\sigma_{l}\left(\text{x}\right)=C_{l}{\left(CV\left(\text{x}\right)\right)}^{2},$$

where *C*_*l*_ is a suitable constant depending on the characteristic mesh size *h* of the FE mesh and *CV*(**x**) denotes the norm of the conduction velocity vector. In order to maintain the anisotropy ratio, we model

$$\sigma_{t,n}\left(\text{x}\right)=C_{l}{\left(CV\left(\text{x}\right)\right)}^{2}1_{CV\left(\text{x}\right)<0.4}+C_{t,n}1_{CV\left(\text{x}\right)\geq 0.4},$$

where a threshold of 0.4 m/s has been considered for the transversal CV, based on the measurements reported in Ferrer et al. (2015). In this way, it is possible to describe in a continuous manner the structural tissue modifications covering all the scales among dense fibrosis and non-fibrotic tissue areas: this allows to capture not only the border zone variability, but also the natural one of the non-fibrotic tissue. Unlike other studies that have distinguished into fixed macro-regions, our strategy might better highlight the role of heterogeneity both during sinus rhythm and AF.

Regarding the effect of electrical remodeling on the ionic channels, we choose to modify the ionic properties in regions characterized by slow conductions. In particular, we consider reductions in transient outward current (*I*_to_), ultrarapid delayed rectifier current (*I*_Kur_) and L-type calcium current (*I*_CaL_), as derived from experimental measures in Courtemanche et al. (1999). With respect to the formulation reported in Courtemanche et al. (1998), we model the maximal conductances *g*_to_, *g*_CaL_ and *g*_Kur_ (expressed in nanosiemens per picofarad) as heterogeneous coefficients, defined by the following laws:

$$\begin{array}{l} {\;g}_{\text{to}}\left(\text{x}\right)=\left(0.5+I_{CV}\left(\text{x}\right)0.5\right){\bar{g}}_{\text{to}},\\ g_{\text{CaL}}\left(\text{x}\right)=\left(0.3+I_{CV}\left(\text{x}\right)0.7\right){\bar{g}}_{\text{CaL}},\\ g_{\text{Kur}}\left(\text{x}\right)=\left(0.5+I_{CV}\left(\text{x}\right)0.5\right){\bar{g}}_{\text{Kur}}, \end{array}$$

where, ${\bar{g}}_{\text{to}}$, ${\bar{g}}_{\text{CaL}}$ and ${\bar{g}}_{\text{Kur}}$ are the homogeneous maximal conductances defined in Courtemanche et al. (1998). *I*_*CV*_(**x**) is a continuous function based on the values of the conduction velocity *CV* going from the non-fibrotic tissue (*I*_*CV*_(**x**) = 1 for all **x** with *CV*(**x**) ≥ 1.25 m/s) to the fibrotic area (*I*_*CV*_(**x**) = 0 for all **x** with *CV*(**x**) ≤ 0.25 m/s). We consider *I*_*CV*_(**x**) = *CV*(**x**)−0.25 for all **x** with 0.25 m/s ≤ *CV*(**x**) ≤ 1.25 m/s.

## 6. Numerical simulations

In this section, we report the numerical results obtained starting from two different CV maps, which are computed from a paroxysmal and a persistent activation map, respectively. The study was divided into a first phase of code validation, followed by the simulation of atrial fibrillation scenarios. In this second phase, we varied the trigger activity location (left superior pulmonary vein and right superior pulmonary vein) and we modified both the electrical and conduction properties to describe the progression of AF remodeling.

Numerical simulations have been performed on the iHEART cluster (Lenovo SR950 8x24-Core Intel Xeon Platinum 8160, 2100 MHz and 1.7TB RAM) at MOX-Department of Mathematics, Politecnico di Milano. The numerical methods presented in this paper have been implemented in life^X^^1^, a high-performance C++ library developed within the iHEART project^2^ and based on the deal.II Finite Element core (see Arndt et al., 2020a,b).

### 6.1. Feeding the Model With Patient-Specific Data

**Figure 3:**
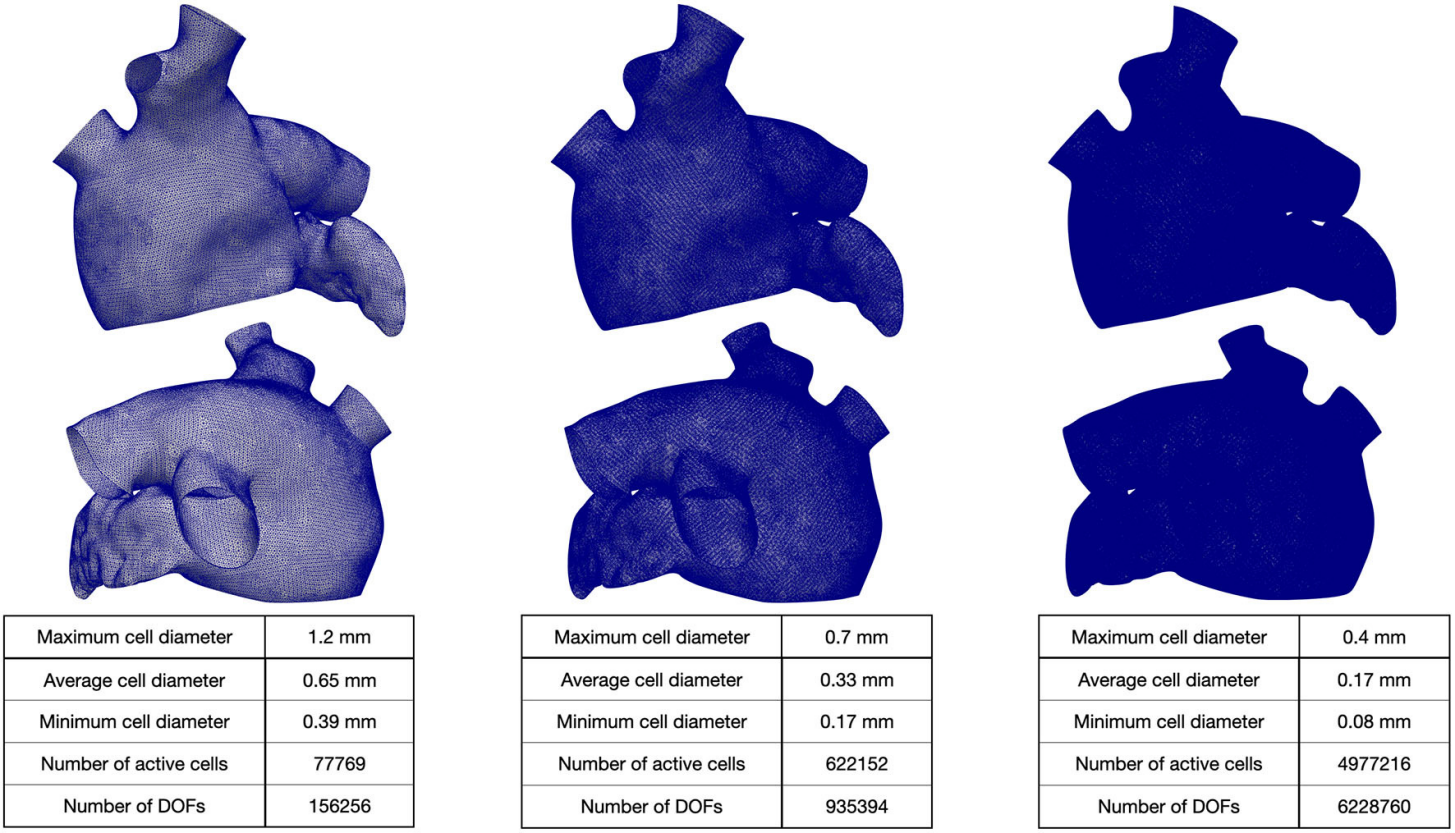
Hexahedral meshes with different levels of refinement. The geometry was obtained from Zygote solid 3D heart model (Zygote Media Group Inc., 2014).

We generated a reference LA geometry by extruding the endocardial surface of the LA given by the Zygote solid 3D heart model Zygote Media Group Inc. (2014). Then, we generate three 3D tagged hexahedral meshes $T_{h}$ of the computational domain. The refined meshes were obtained by recursively splitting each hexahedral element in eight elements (main features of the computational meshes are reported in Figure 3).

Then, we applied the atrial Laplace Dirichlet rule based method (LDRBM) developed by Piersanti et al. (2020) to mathematically reconstruct the distribution of myocardial fibers, which direct the electric potential propagation in the cardiac tissue. For the diffusivity tensor, we consider two configurations:

1. $C_{l}=3.0\cdot 10^{-4}\text{s}$ and $C_{t,n}=0.48\cdot 10^{-4}\text{s}$ to approximatevely mimic the patient-specific behavior (coefficients were tuned on a simplified geometry by Piersanti et al. (2020) to numerically match the conduction velocities);
2. $C_{l}=2.0\cdot 10^{-4}\text{s}$ and $C_{t,n}=0.32\cdot 10^{-4}\text{s}$ to reproduce a condition of increased slow conduction with respect to the given parametrization, which potentially represents the progression of the disease.

**Figure 4:**
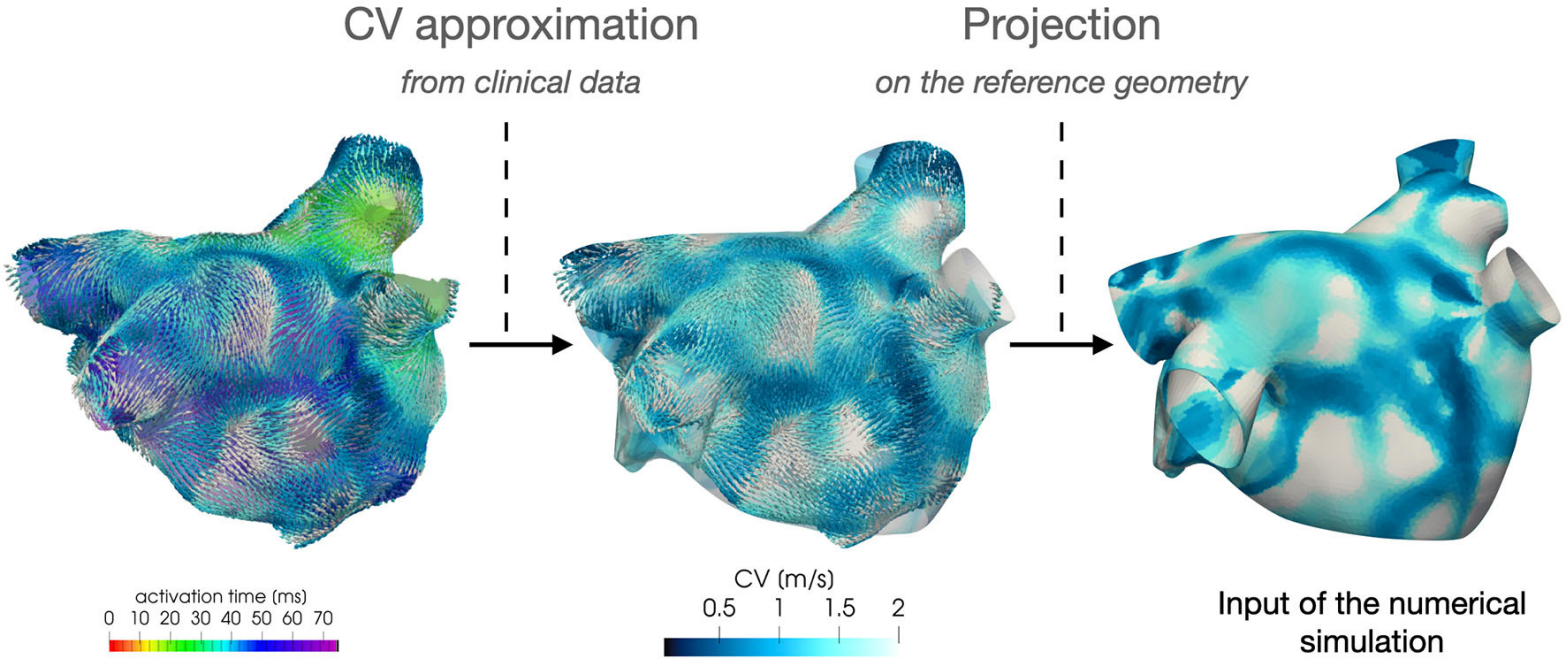
Feeding the model with data: numerical approximation of CV field from patient activation map and its projection onto the computational geometry.

**Figure 5:**
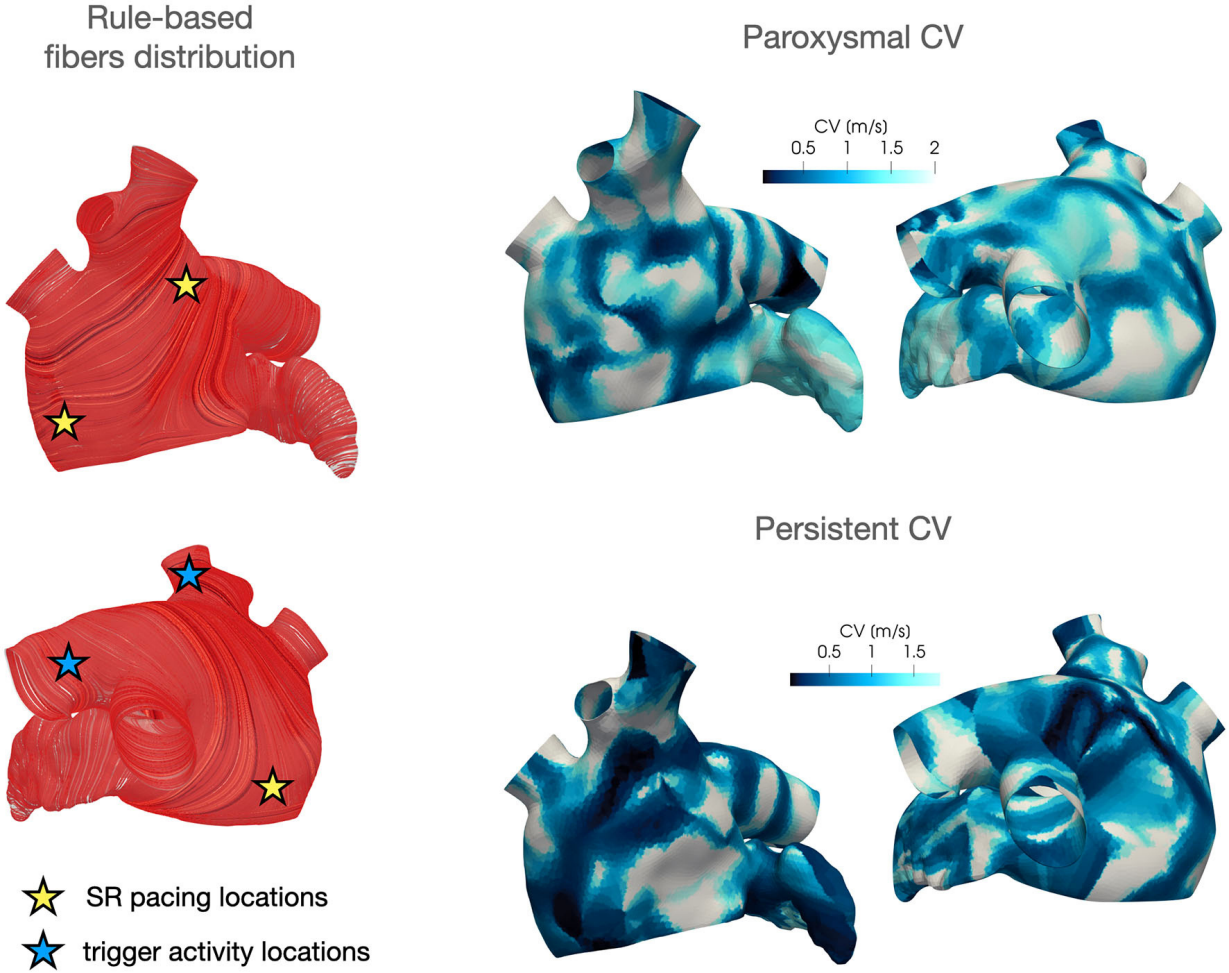
LDRBM fibers distribution and projected CV field for the two patients considered in this work.

Regarding the parametrization of the diffusivity tensor and of the ionic properties, we consider two *CV*(**x**) fields based on clinical data of two AF patients, classified as paroxysmal and persistent, respectively. Each CV field is approximated, following the procedure described in section 4, from the activation map in SR acquired by the Arrhythmia Unit at San Raffaele Hospital. After manually aligning the maps and the Zygote geometry, we project the numerically approximated conduction velocities onto the Zygote mesh using nearest-neighbor interpolation. This pipeline is reported in Figure 4. The final heterogeneous coefficients *CV*(**x**) for both cases, along with LDRBM fibers distribution, are reported in Figure 5.

The projection of the CV data onto the Zygote geometry might introduce a non-negligible geometrical error, limiting the ability of the model in reproducing the patient-specific behavior. However, we expect that this approximation has a non-significant impact on the model's ability to reproduce the mechanisms of atrial fibrillation, as the result of the functional and structural factors described in section 2.

The mapping of CV data onto the reference geometry can be performed also using an alternative projection strategy, based on the universal atrial coordinates, developed in Roney et al. (2019a).

### 6.2. Sinus Rhythm Activation

For numerically simulating sinus rhythm activation, we employ three spherical impulses of radius *r*_*p*_ = 6 mm, an amplitude of 200s^−1^ and a duration *t*_*app*_ = 5 ms.

In the spirit of calculation verification (see Viceconti et al., 2020), we assess the accuracy of the discretization scheme in approximating the activation pattern. Specifically, we compare the activation times resulting from the post-processing of the numerical solutions related to different choices of (*h*, Δ*t*), with *h* = {0.65, 0.33, 0.17}mm and Δ*t* = {0.1, 0.05, 0.025} ms. Here, the unipolar activation map (*AT*) at each point **x** of the computational mesh is computed as the time of maximum variation of the transmembrane potential approximation, i.e.,

$$AT_{\left(h,\Delta t\right)}\left(\text{x}\right)={\arg\max}_{n=0,\ldots ,N_{t}-1}\left|\frac{\alpha_{\text{BDF}}{\text{u}}_{h}^{n+1}\left(\text{x}\right)-{\text{u}}_{h,\text{BDF}}^{n}\left(\text{x}\right)}{\Delta t}\right|,$$

where ${\text{u}}_{h}^{n+1}\left(\text{x}\right)$ stands as the approximation of the transmembrane potential *u*(*without*_*h*_) at point **x** and time *t*^*n*+1^. To compare the numerical results, we compute the relative error with respect to the activation map computed using *h*_3_ = 0.17mm and Δ*t*_3_ = 0.025ms, that is:

$$err\left(h_{i},\Delta t_{i}\right)=\frac{||AT_{\left(h_{i},\Delta t_{i}\right)}\left(\text{x}\right)-AT_{\left(h_{3},\Delta t_{3}\right)}\left(\text{x}\right){||}_{1}}{||AT_{\left(h_{3},\Delta t_{3}\right)}\left(\text{x}\right){||}_{1}},$$

where the norm 1 is defined as follows:

$$||AT_{\left(h_{3},\Delta t_{3}\right)}\left(\text{x}\right){||}_{1}=\underset{\text{x}\in T_{h_{3}}}{\sum }|AT_{\left(h_{3},\Delta t_{3}\right)}\left(\text{x}\right)|.$$

**Figure 6:**
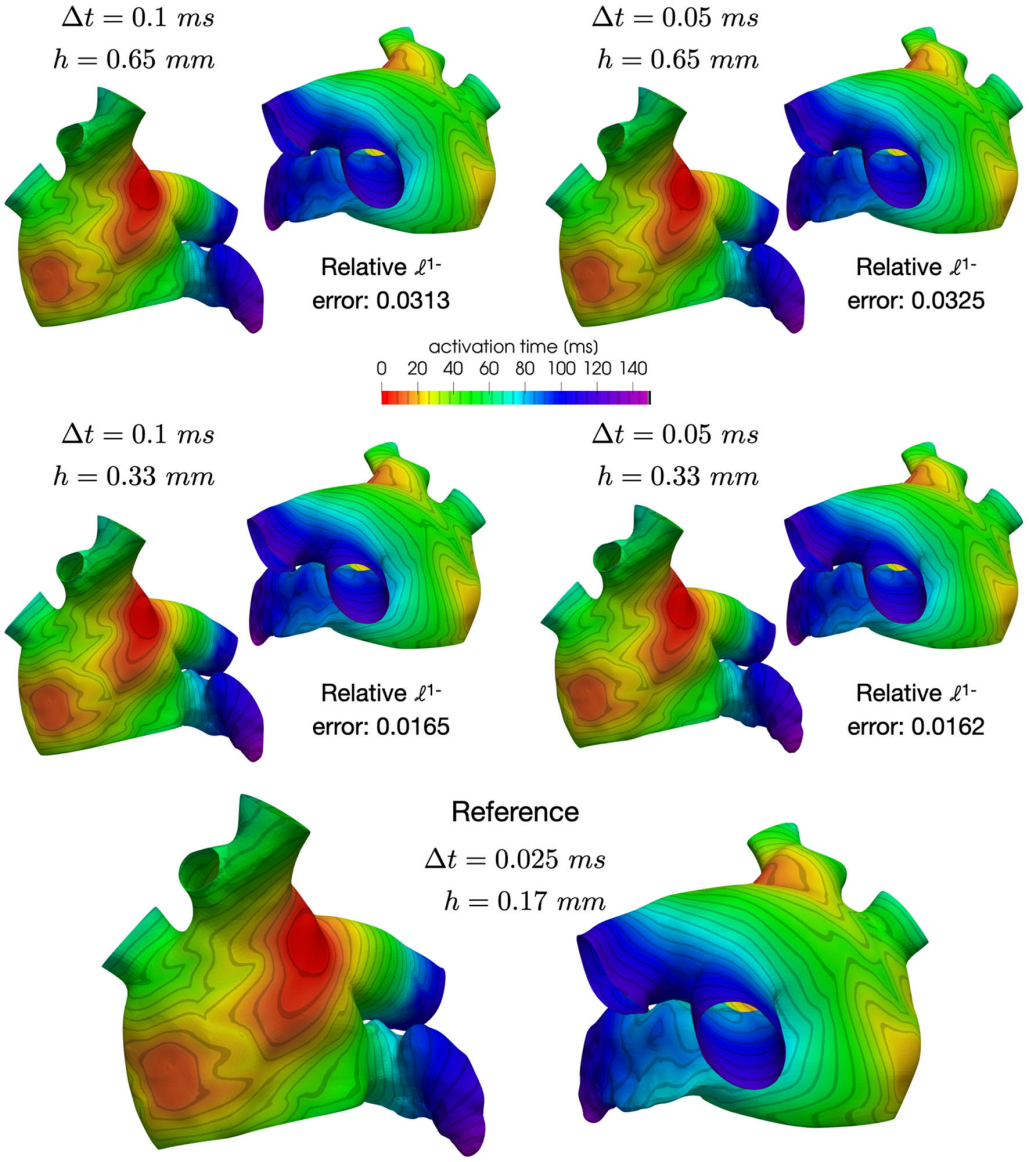
Comparison of activation maps numerically approximated using different space and time discretizations.

The mesh with 1-level refinement, with an average diameter *h*≈0.33mm, captures all the space scales of the electrophysiological problem in both cases, with a relative error of 0.016 with respect to the mesh with 2-levels refinements (see Table 1). Activation maps reported in Figure 6 are not distinguishable in norm 1. This motivates the use of a discretization with (*h*, Δ*t*) = (0.33mm, 0.05ms) for all the numerical simulations presented in the following sections.

**Table 1:** Relative error *err*(*h*, Δ*t*) in approximating the activation time for different choices of (*h*, Δ*t*).

	**(0.65mm,** **0.1ms)**	**(0.65mm,** **0.05ms)**	**(0.33mm,** **0.1ms)**	**(0.33mm,** **0.05ms)**
PAF	0.0313	0.0325	0.0165	0.0162
PsAF	0.0314	0.0311	0.0169	0.0157

### 6.3. Persistent AF

In these numerical simulations, we adopt the CV field obtained by projecting the persistent patient conduction velocity map onto the Zygote geometry (see Figure 5, bottom-right). We consider *T* = 6 s as final time of all the simulations reported in this section.

For the baseline activation, we employ three spherical impulses of radius *r*_*p*_ = 6 mm, an amplitude of 200 s^−1^ and a duration *t*_*app*_ = 5 ms, with a baseline frequency of 1.82 Hz (which corresponds to 109 bpm in physiological conditions). We superimpose a trigger activity, which is either located in the left superior pulmonary vein or in the right superior pulmonary vein (see Figure 5, left), modeled as one cubic impulse of length *r*_*p*_ = 6 mm, with an amplitude of 200 s^−1^ and a duration *t*_*app*_ = 5 ms and a high frequency of 8.26 Hz, which is derived from clinical measurements.

**Figure 7:**
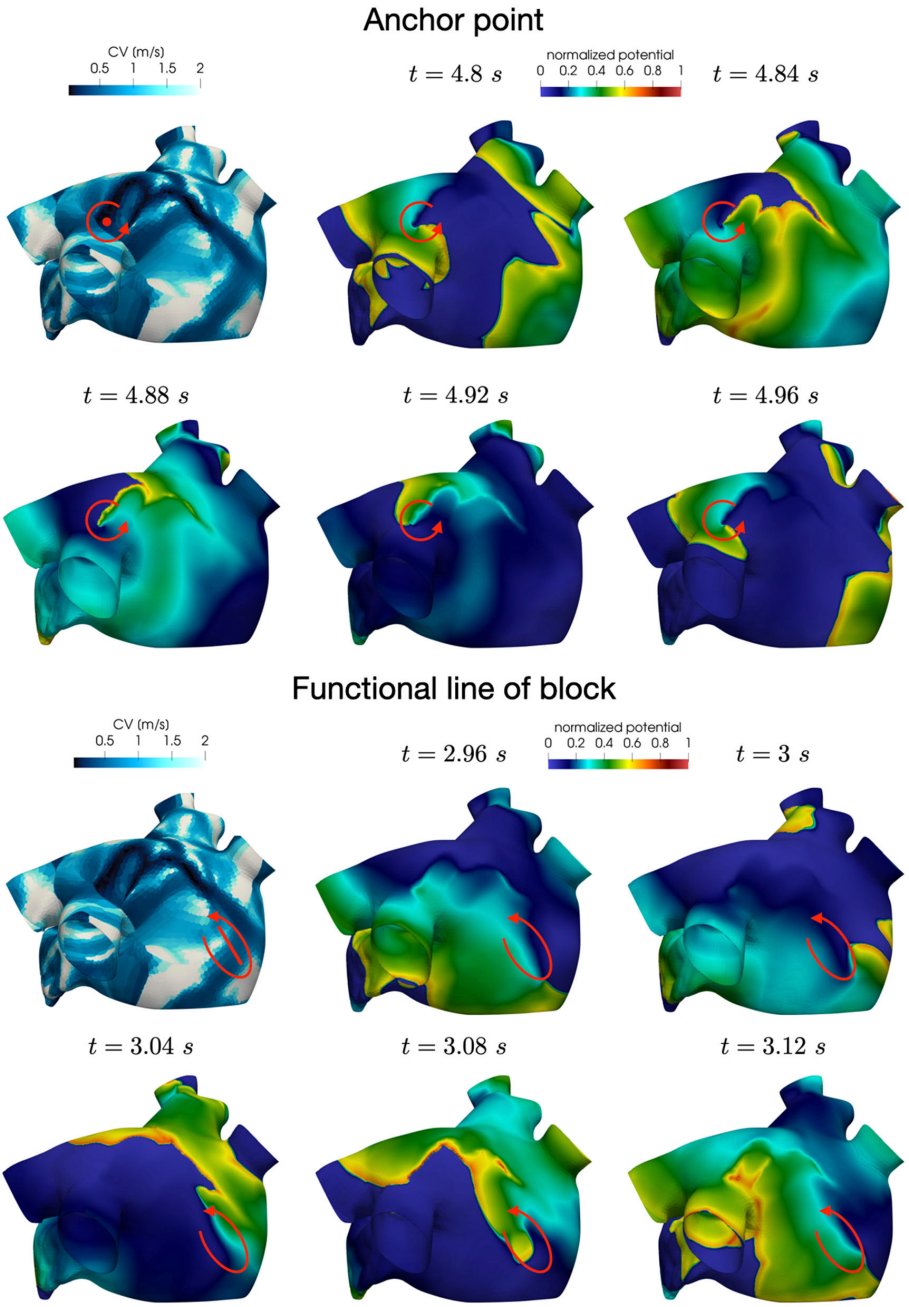
Persistent AF: examples of two localized reentries with different anchoring sites. An anchor point, located in an area of severe slow conduction, allows rotor's stabilization **(top)**. Tissue heterogeneity, instead, force the rotor to travel along a functional line of block **(bottom)** .

In all the simulations, the high-frequency trigger activity becomes preponderant with respect to the low-frequency baseline activity. However, the interplay between the continuous triggering from one of the pulmonary vein and the baseline activation generates instabilities that lead to the formation of localized reentries. As the triggering point changes, the induction of the phenomenon always occurs within a few seconds from the start of the simulation. The instabilities then propagate along the tissue forming localized reentrant circuits (see Figure 7).

**Figure 8:**
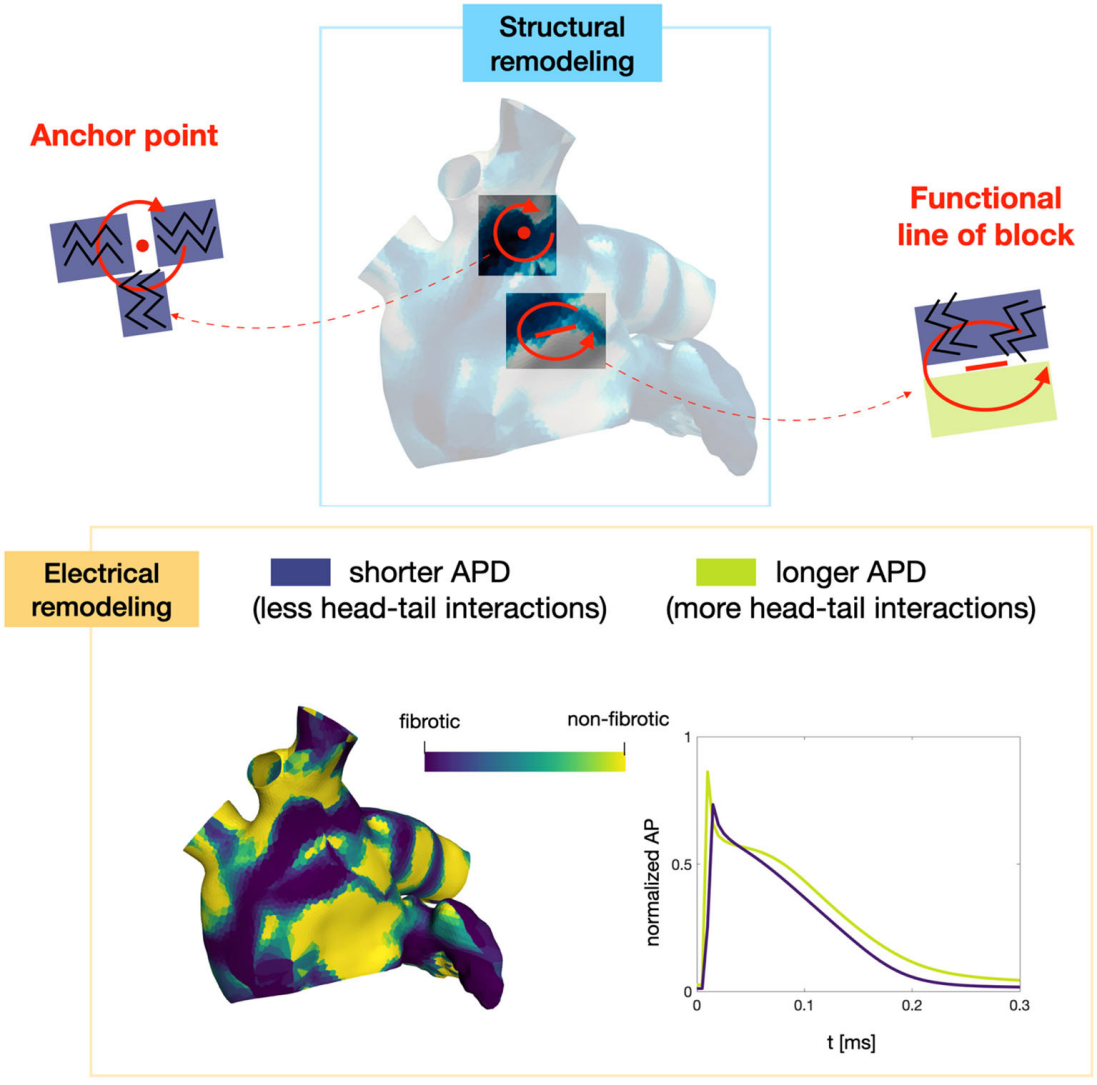
Role of the electrical and structural remodeling in the formation of localized reentries. The formation of functional lines of block occurs in areas of heterogeneous tissue, where the wavefront is likely to meet its tail due to conduction and APD heterogeneity. In areas of severe slow conduction, the rotor stabilize thanks to the low wavefront's speed and the short APD.

Numerical simulations confirm that, in persistent AF, localized reentries can be anchored to areas of severe slow conduction, which are a distinctive feature of this group of patients. In Figure 7, we observe the formation of anchor sites, under either the form of points (top) or functional lines of block (bottom), around which the wavefront rotates. The shape of these anchor sites can be directly related to the properties of both the substrate and the ionic species, subject to electrical remodeling (see Figure 8). In the case of localized reentries that take place around functional lines of block, the rotor is not anchored to a point but travels along this line. The formation of these lines occurs in areas of heterogeneous tissue, where the wavefront meets its tail, forcing the rotor to travel along this functional block. The length of these lines is naturally linked to the conductive and refractory properties of the tissue: areas with physiological conduction velocities have a longer APD duration, which increases the probability of forming head-to-tail interactions, thus forcing a lengthening of the line. On the contrary, in areas of severe slow conduction, the rotor tends to stabilize thanks to the combination of two effects: the wavefront slowly travels the circuit and the shorter APD decreases the possibility of head-tail interaction, due to the local changes in the ionic properties.

We now consider the case with $C_{l}=2.0\cdot 10^{-4}\;\text{s}$ and $C_{t,n}=0.32\cdot 10^{-4}\;\text{s}$ to reproduce a condition of increased slow conduction with respect to the previous results. In addition, we also introduce an increased electrical remodeling in areas of slow conduction, encoded by the following modifications of the ionic currents parametrization: *I*_*CV*_(**x**) = (*CV*(**x**)−0.5)/0.75 for 0.5 m/s ≤ *CV*(**x**) ≤ 1.25 m/s is a continuous function linking the non-fibrotic tissue ( *I*_*CV*_(**x**) = 1 for all **x** with *CV*(**x**) ≥ 1.25 m/s) to the fibrotic area (*I*_*CV*_(**x**) = 0 for all **x** with *CV*(**x**) ≤ 0.5 m/s).

**Figure 9:**
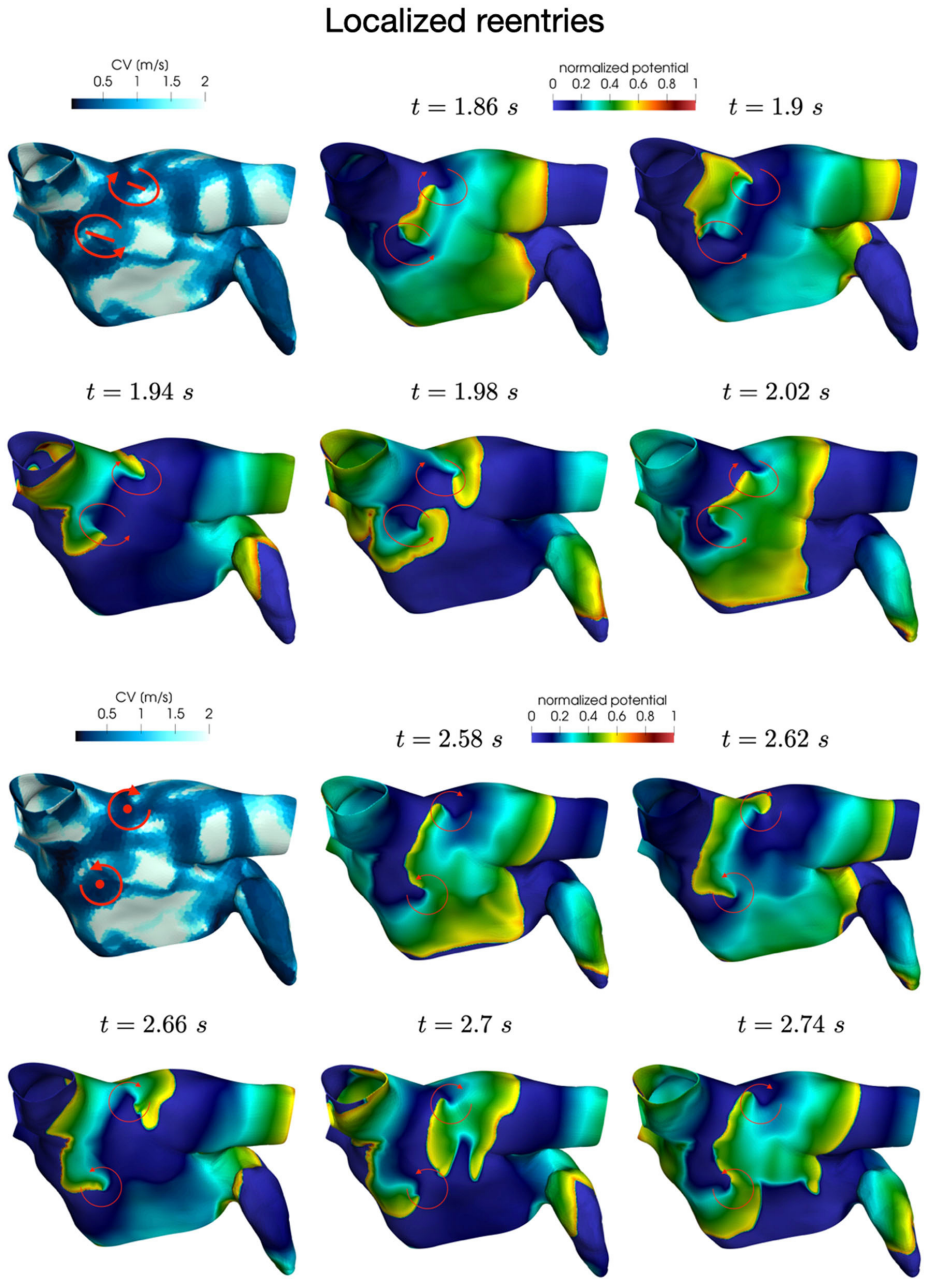
Anchor points stabilization in area of severe slow conduction and enhanced electrical remodeling.

Also in these simulations, the induction of localized reentrant circuits occurs within a few seconds from the beginning of the simulation. Compared to the previous case, we observe an increase of anchor points, which are also more stable than the previous ones. That is, the signal rotates around these points for a longer period of time, and the arrival of other wavefronts is less likely to interrupt the reentrant circuit. Numerical results in Figure 9 show how the unstable circuits that travel along functional lines of block (top), rapidly stabilized in anchor points in an area of severe slow conduction (Figure 9, bottom).

**Figure 10:**
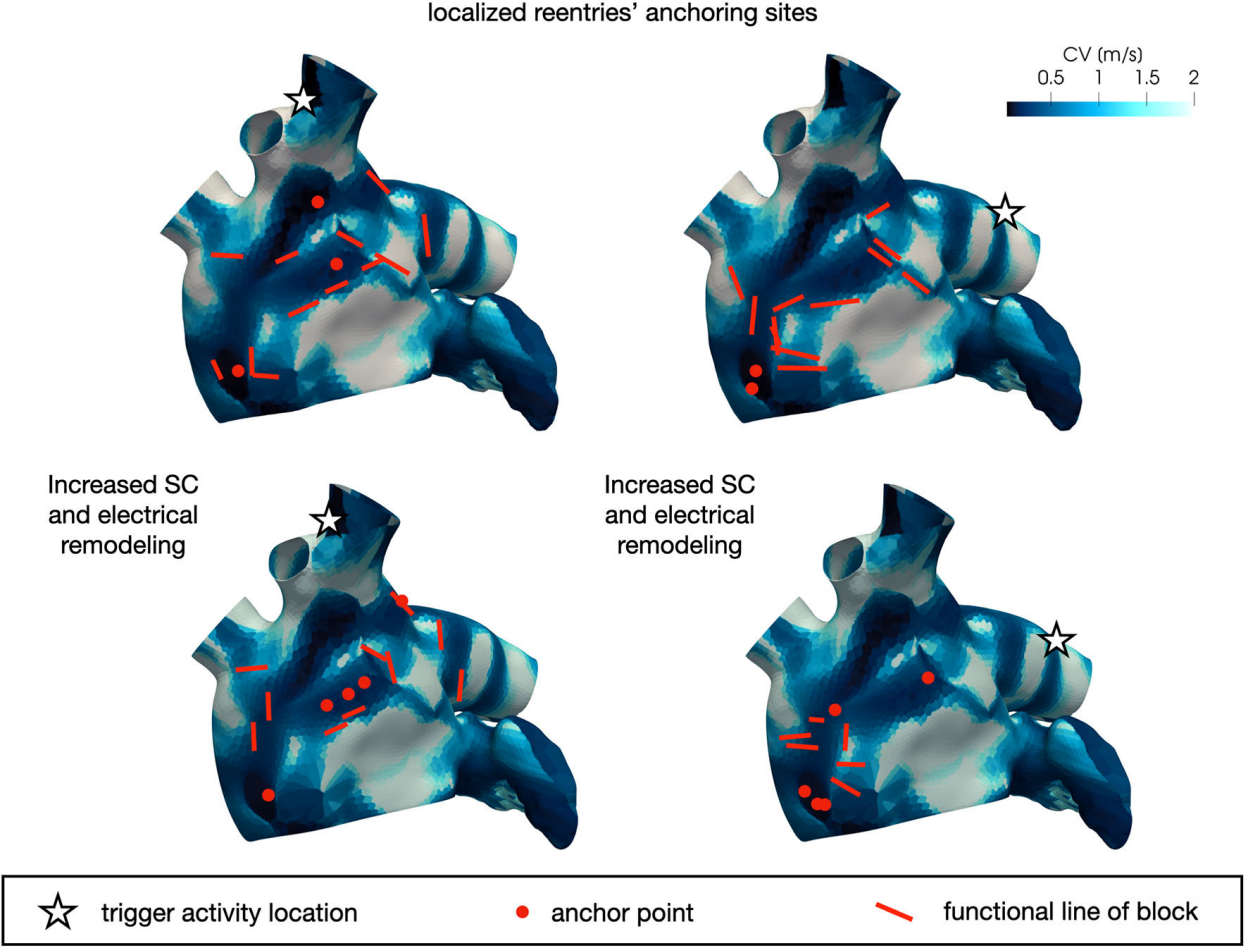
Position of localized reentry anchor points or functional lines of block in the analyzed cases generated from the CV field of the persistent patient. The progression of the disease (bottom) generates more anchor points, which sustain the localized reentries.

In Figure 10 we display the anchor areas that arise during the numerical simulations. We compare the triggering activity from the left or right pulmonary vein (right column and left column, respectively) and a condition of increased slow conduction and more severe electrical remodeling with respect to the starting one (second row vs. first row). As pointed out previously, anchor points are clustered in areas of severe slow conduction, while functional lines of block form in areas with heterogeneity in conduction. The progression of the disease, with increased slow conduction and more severe electrical remodeling, generates more anchor points with respect to the previous case, which might be the drivers preserving the persistence of the AF event.

Our results show that in a patient suffering from persistent fibrillation, the triggering is responsible for the induction of several localized reentrant circuits, however their sustainment might be motivated by the stabilization of localized reentry in anchor points. This result seems to be able to explain the mechanism behind the persistence of the phenomenon.

### 6.4. Paroxysmal AF

In these simulations, we adopt the CV field obtained by projecting the paroxysmal patient conduction velocity map onto the Zygote geometry (see Figure 5, top-right). We consider *T* = 10 s as final time of all the simulations reported in this Section.

Regarding the activation sequence, we used the same parameters of the previous test case, with the exception of the radius of the baseline activation increased to *r*_*p*_ = 7 mm. The main differences in the parametrization are due to the different *CV* field, which affects the diffusivity tensor, expression of a different electrical substrate, and the electric remodeling areas. Here, the mean conduction velocity is approximatively 40 cm/s higher than the previous case (with a comparable standand deviation).

**Figure 11:**
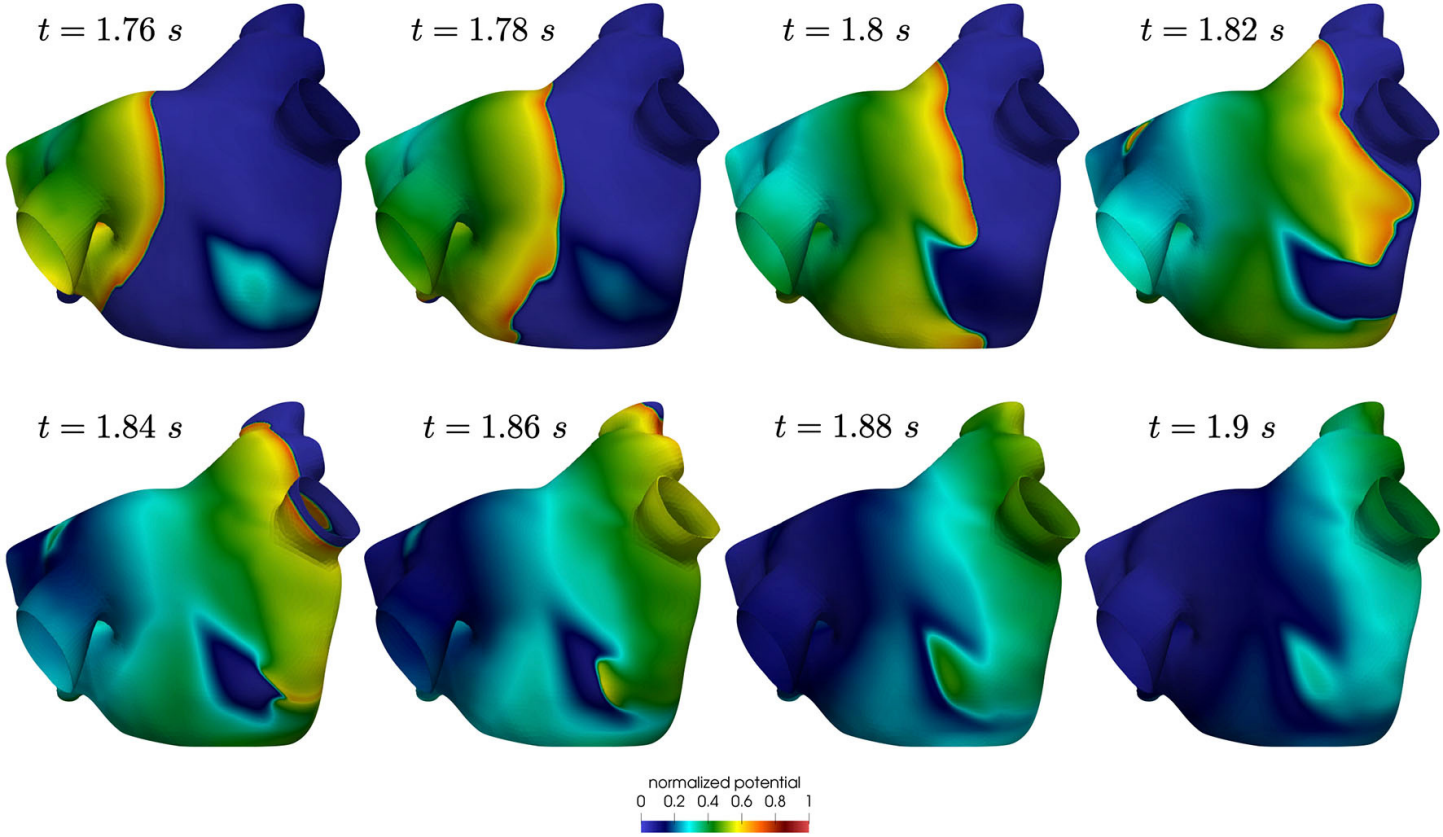
Non sustained localized reentry in paroxysmal AF.

Also in this case, the high-frequency trigger activity becomes preponderant with respect to the low-frequency baseline activity. The interplay between the continuous triggering from one of the pulmonary vein and the baseline activation generates instabilities that lead to the formation of localized reentries. However, the induction of localized reentries occurs less frequently than in the previous case, and very often instabilities create reentries that terminate quickly due to head-tail interactions, as shown in Figure 11.

**Figure 12:**
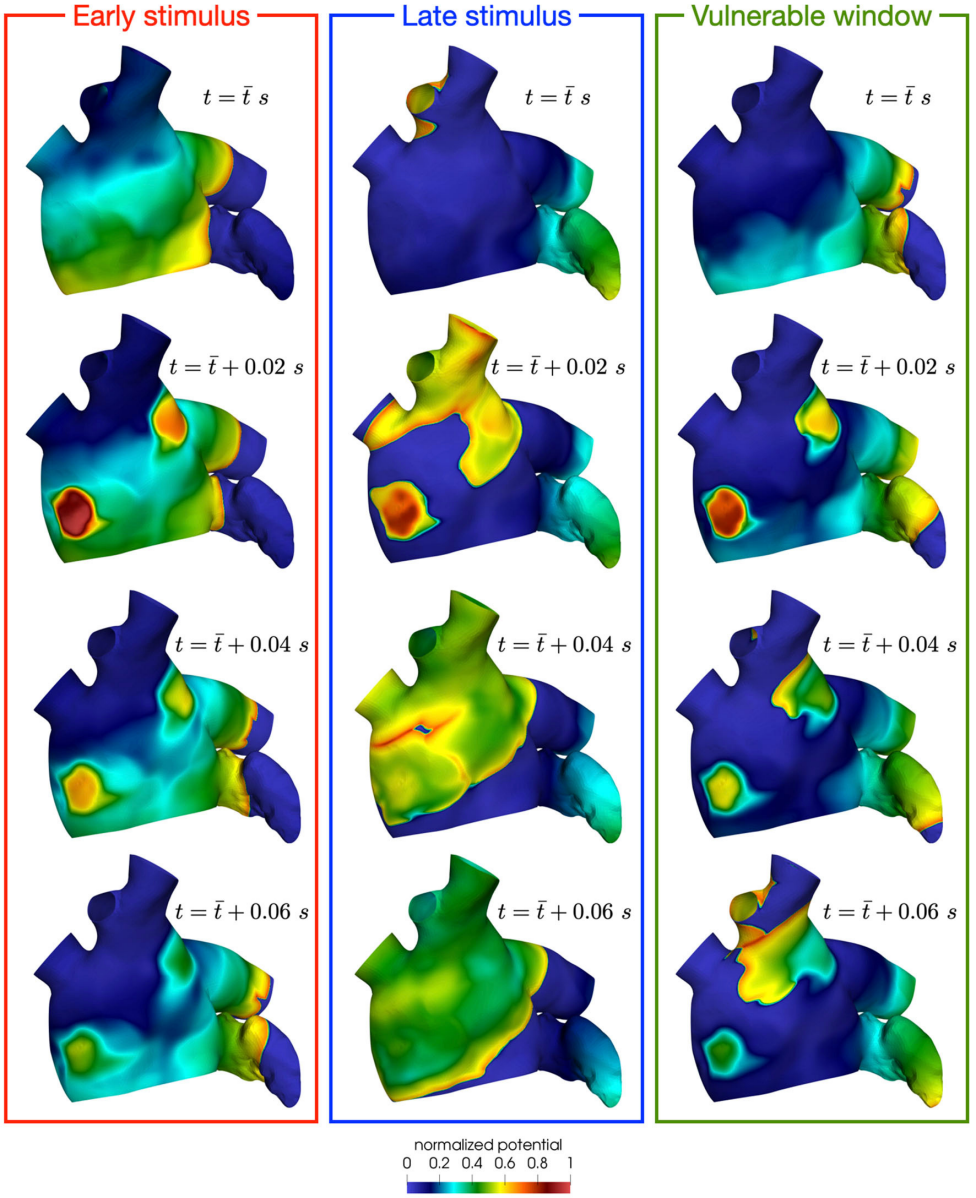
Effect of a stimulus delivered before **(left)**, after **(center)** or in the vulnerable window **(right)**. Only in the latter case, the instability creates rotors that might form reentrant circuits.

The mechanisms of induction of localized reentries can be explained using the pinwheel experiment proposed by Winfree and reviewed in Karma (2013). This experiment is based on the interplay between a first propagating wavefront generated by a first stimulus (S1) and a second stimulus (S2). The time interval between the two stimuli (S1–S2), the dimension of the second stimulus, the refractory properties of the tissue and the conduction speed of the tissue are factors that contribute to the so-called vulnerable window. If S2 is delivered in this window, it creates an instability with refractory tissue, resulting in one or two rotors that might form a reentrant circuits (see Figure 12, right). If the S2 is delivered outside the vulnerable window, there are two possible scenarios: early stimulus, which means that S2 is applied when the tissue is not re-excitable yet (see Figure 12, left); late stimulus, which results in a single extra beat without localized reentries formation (see Figure 12, center). In this paroxysmal case, the higher conduction speed significantly reduces the vulnerabile window, thus lowering the probability of reentry formation. On the contrary, in a condition of reduced conduction speed, such as the persistent one, the vulnerable window widens. This also corresponds to a condition of fewer head-tail interactions, in which the sustaining of localize reentry is more likely, as we have observed from the previous results.

**Figure 13:**
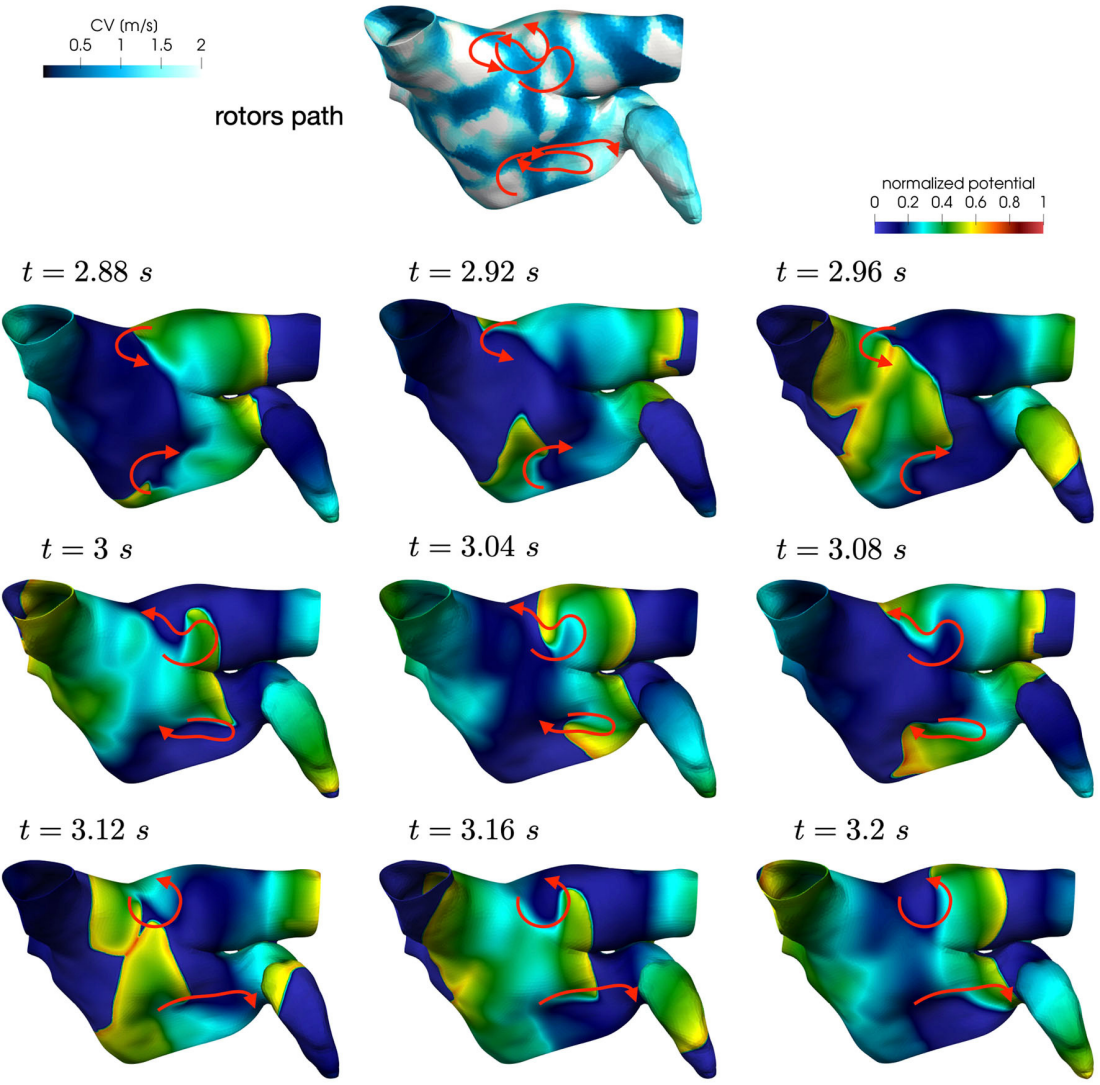
Rotors position in unstable localized reentry in paroxysmal AF.

**Figure 14:**
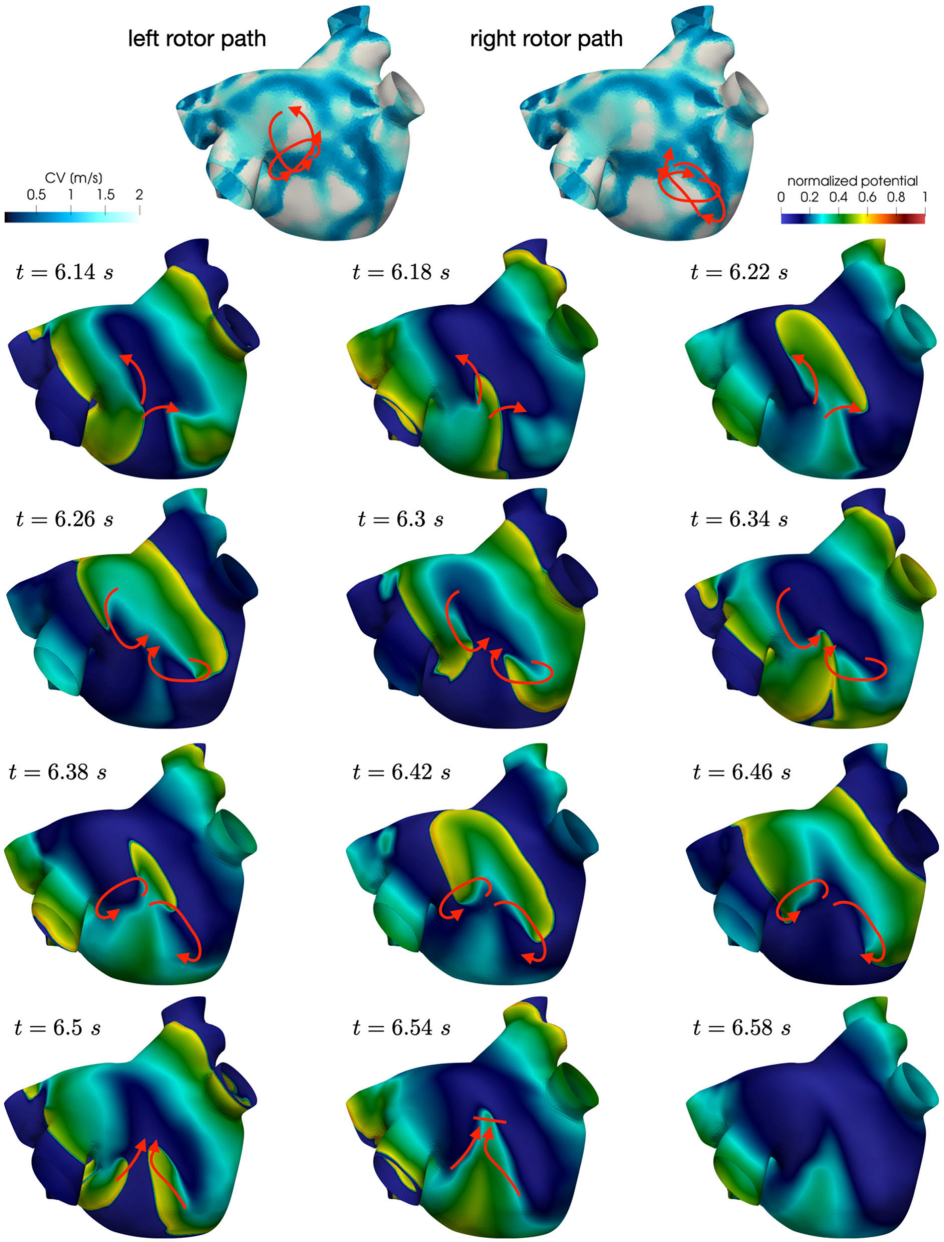
Rotors position in unstable localized reentry in paroxysmal AF. The reentry is interrupted by head-tail interaction.

We now consider the case with $C_{l}=2.0\cdot 10^{-4}\;\text{s}$ and $C_{t,n}=0.32\cdot 10^{-4}\;\text{s}$ to reproduce a condition of increased slow conduction with respect to previous results. In addition, we also introduce an increased electrical remodeling in areas of slow conduction, encoded by the following modifications of the ionic currents parametrization: the values of *I*_*CV*_(**x**)(*CV*(**x**)−0.5)/0.75 for 0.5 m/s ≤ *CV*(**x**) ≤ 1.25 m/s range from the non-fibrotic tissue (*I*_*CV*_(**x**) = 1 for all **x** with *CV*(**x**)≥1.25 m/s) to the fibrotic area (*I*_*CV*_(**x**) = 0 for all **x** with *CV*(**x**) ≤ 0.5 m/s). Only in this configuration, when the trigger is in the left pulmonary vein, we observe the formation of unstable localized reentries sustained for a few seconds (partially represented in Figures 13, 14).

Numerical simulations show that in paroxysmal AF localized reentries cannot anchor to areas of severe slow conduction, which are absent in this group of patients. In Figure 13, we observe the dynamics of a wandering rotor along mild slow conduction areas. The movement of the rotor is dictated by head-tail interactions that occur along the functional line of block. In this case, the higher conduction velocity causes the signal to reach its tail and, being unable to continue along the same reentrant circuit, it deviates by moving its rotor along the tissue. The absence of areas of severe slow conduction prevent the anchoring of the rotor, by making the wavefront dynamics unstable and therefore more likely to be interrupted (see Figure 14).

## 7. Limitations of the study

MRI-based models have been adopted for exploring the link among localized reentry locations and fibrosis distribution. Starting from this correlation, Boyle et al. (2019) developed a new ablation strategy, targeting regions with a high probability of reentry anchoring. However, it is not possible to deduce the electrophysiological properties of a patient from the imaging alone, even if relationships between variations in speed and pixel intensity have recently been shown in Aronis et al. (2020). This approach, although able to identify the structural causes of AF, could limit the model in reproducing the so-called functional causes, i.e. those resulting from the dynamics of the transmembrane potential. Such functional causes modify the localized reentry locations, as shown in the AF persistent case in Deng et al. (2017), potentially compromising the ablation target identification based exclusively on structural data, especially at the early stages of AF. For this reason, we consider an approach based on direct measurements of the electrophysiological properties from electroanatomical mapping, as also done in Corrado et al. (2018) and Lim et al. (2020). In this way, we encode in the model patient-specific AF factors such as electrophysiological heterogeneity (together with structural heterogeneity, consistently).

Following Courtemanche et al. (1999), we consider a minimal remodeling of the ionic currents in slow conduction areas involving *I*_to_, *I*_Kur_ and *I*_CaL_. Nevertheless, cellular remodeling in human hearts affected by AF results in modifications of additional ionic currents, such as the inward rectifier potassium current (*I*_K1_) and the sodium current (*I*_Na_), as reported in Bosch et al. (1999), Workman et al. (2001), and Zahid et al. (2016), which may contribute to the functional modifications of localized reentries.

Since we worked with mapping data acquired on the endocardium, we do not take into account parameters such as atrial wall thickness heterogeneities, which can lead to dissociation among layers in the atrial wall, as reported in Gharaviri et al. (2020). Anisotropy ratio cannot be identified from the sinus rhythm map alone, but requires the acquisition of additional data, such as paced maps, as shown in Roney et al. (2019b). This absence of additional information is surrogated in our model by a rule-based model of the fibers, which reconstructs the basic architecture of the conduction, as done also in MRI-based models.

These limitations should be addressed to construct reliable patient-specific models, which can potentially identify targets of ablation. Since the focus of our work is related to the role of heterogeneity in conduction and electrical remodeling in the formation and sustainment of localized reentries, we believe that the impact of these limitations on our main findings is limited. The possibility of integrating a larger number of information, coming from the mapping systems themselves (geometry, pacing maps and endocavitary signals) or from imaging would improve the model ability of reproducing increasingly realistic scenarios.

## 8. Conclusion

The present study develops a new parametrization of the monodomain equation coupled with the CRN ionic model based on conduction velocity data with the aim of providing insights about the role of the electrical substrate that triggers and sustains AF. In the numerical tests that have been performed, significant differences emerged in the comparison between a substrate characterized by the presence of severe slow conductions, typical of patients with persistent AF, and a substrate with less severe slow conductions (and on average higher conduction velocity), typical of patients with paroxysmal AF.

In our numerical results, we observed the induction of several localized reentries in the persistent case in all the tested conditions. Conversely, the induction of the localized reentries occurs less frequently in the paroxysmal case, due to a reduction of the vulnerable window. Moreover, several of these localized reentries are not self-sustained, since they terminate due to head-tail interactions.

In the persistent AF case, we observed the formation of anchoring areas, in the form of anchor points in areas of homogeneous severe slow conduction or functional lines of block where the tissue is heterogeneous. We also observed that a greater severity of slow conduction corresponds to the formation of more stable anchor points. In the paroxysmal AF case, we notice that the triggering from the pulmonary vein does not lead to anchor points that sustain fibrillation, but forms unstable wandering rotor along mild slow conduction areas. From these numerical results, we can associate the progression of the disease with a stabilization of localized reentries in slow conducting areas.

These results indicate that our new model parametrization has the potential to be used for patient-specific simulations of both paroxysmal and persistent AF in the LA. Furthermore, the numerical simulation of localized reentries might provide indications on the electrophysiological substrate, that might contribute to the identification of ablation targets.

## Data Availability Statement

The original contributions presented in the study are included in the article, further inquiries can be directed to the corresponding author.

## Ethics Statement

The studies involving human participants were reviewed and approved by Ethical Committee on Human Research, San Raffaele Hospital, Milan, Italy. The patients/participants provided their written informed consent to participate in this study.

## Author Contributions

SP, LD, and MS designed the method. SP realized the numerical simulations. AF and LL analyzed the data and their interpretations. All authors discussed the numerical results. SP wrote the manuscript. All authors edited the manuscript.

## Conflict of Interest

AF and PD have received consultant fees from Boston Scientific. The remaining authors declare that the research was conducted in the absence of any commercial or financial relationships that could be construed as a potential conflict of interest.

## Footnotes

^1^https://lifex.gitlab.io/lifex.

^2^iHEART - An Integrated Heart Model for the simulation of the cardiac function, European Research Council (ERC) grant agreement No 740132, P.I. Prof. A. Quarteroni.
